# Validity of a Convolutional Neural Network-Based, Markerless Pose Estimation System Compared to a Marker-Based 3D Motion Analysis System for Gait Assessment—A Pilot Study

**DOI:** 10.3390/s25216551

**Published:** 2025-10-24

**Authors:** Korbinian Ksoll, Rafael Krätschmer, Fabian Stöcker

**Affiliations:** 1 OPED GmbH, 83626 Valley/Oberlaindern, Germany; 2 Prevention Center, Department Health and Sport Sciences, TUM School of Medicine and Health, Technical University of Munich, 80809 Munich, Germany

**Keywords:** convolutional neural network (CNN), markerless pose estimation, 3D motion capture, orthelligent vision, gait analysis, gait variability, concordance correlation coefficient (CCC), coefficient of variation (CV)

## Abstract

Gait analysis is a valuable tool for a wide range of clinical applications. Until now, the standard for gait analysis has been marker-based 3D optical systems. Recently, markerless gait analysis systems that utilize pose estimation models based on Convolutional Neural Networks (CNNs) and computer vision have gained importance. In this pilot study, we validated the performance of a CNN-based, markerless pose estimation algorithm (Orthelligent^®^ VISION; OV) compared to a standard marker-based 3D motion capture system in 16 healthy adults. Standard gait metrics were analyzed by calculating concordance correlation coefficients (CCCs) and coefficients of variation. With regard to gait event detection, we found good overlaps for both systems. Compared to the marker-based motion analysis, OV achieved a strong to almost complete concordance regarding the sagittal measurement of cadence, gait variability, step time, stance time, step length, and double support (CCC ≥ 0.624), as well as regarding the frontal plane parameters of cadence, step time, stance time, and step width (CCC ≥ 0.805). For gait symmetry only, we found a moderate to weak correlation. These results support the CNN-based, markerless gait analysis system OV as an alternative to marker-based 3D motion capture systems for a broad variety of clinical applications.

## 1. Introduction

Clinical gait analysis is a valuable tool for the systematical, objective, and quantitative examination of an individual’s gait and movement patterns during walking [1,2,3]. In clinical practice, gait analysis can offer considerable added value for medical and therapeutic care with a wide range of applications. For example, it can be used in diagnostics to better understand individual causes of gait and movement disorders. Gait analysis can also support the planning and management of treatments according to the patient’s individual condition and abilities [4]. Moreover, compared to visual observation only, gait analysis tools, with their reliable source of data, can serve as a solid basis for treatment decisions while enabling transparent documentation of treatment progress. Its areas of applications range from rehabilitation after orthopedic injuries and surgeries to the monitoring of patients with neurological diseases such as stroke, multiple sclerosis, or Parkinson’s disease [1,2,3,5,6,7,8].

The gold standard in movement and gait analysis is marker-based 3D motion capture using multi-camera systems. These systems require extensive preparation by specialized personnel, specific equipment, and considerable space, which make 3D motion capture time-consuming and expensive. Due to these high demands, 3D motion capture is not accessible for most healthcare professionals although its use offers highly precise movement analysis [9,10]. Recent developments using computer vision technologies based on artificial intelligence (AI) and machine learning (ML) techniques might overcome these barriers. These systems work with a standard 2D camera that records human movement, from which pose estimation algorithms then derive precise kinematic measurements [9]. In principle, pose estimation models use ML algorithms to identify key points and anatomical landmarks of the body such as joints, and analyze their movement patterns to measure gait parameters like walking speed and step length [11]. Convolutional Neural Networks (CNNs) provide the base for these pose estimation models. Their convolutional layers decompose images into feature maps to identify body patterns like edges and structures. In the next step, pooling layers filter the most relevant features. Finally, fully connected layers process the extracted features and classify the image. These CNNs are trained continuously by back-propagation. This means that CNNs use prediction errors to correct their internal parameters while improving their classification abilities. In this way, pose estimation algorithms learn to capture the body’s key landmarks automatically during the prediction phase to calculate joint angles and motion patterns solely based on a standard video recording [10].

It has been shown that these pose estimation algorithms are sufficiently accurate for clinical gait analysis [9,10,12]. Compared to marker-based systems, the pose estimation algorithm MediaPipe showed a good concordance in terms of spatio-temporal gait parameters [9,10,11]. Despite their promising results, the accuracy of current 2D pose estimation algorithms to detect anatomical key points depends on factors like the training of the underlying ML model and the quality of the data source [10,13]. Additionally, the use of pose estimation models still requires certain hardware (e.g., webcam and computer) as well as programming knowledge which still limits the widespread clinical use of gait analysis. Therefore, the development of novel software as medical products offers new possibilities to healthcare professionals. Hereby, the software has to fulfill applicable regulatory requirements, as well as laws on data protection, next to the quality criteria objectivity, validity, and reliability. Further, the software should be inexpensive to purchase and also run with low hardware demands, as the use of tablets allows mobile use. Finally, a markerless gait analysis software needs to be easy to use, with an easy-to-understand report of analysis results [4,12,14,15]. To prove the validity as basis for further research, the aim of this pilot study is to compare spatio-temporal gait parameters measured by a novel software application as a medical device for markerless gait analysis to the gold standard 3D motion capture. In this regard, we hypothesized that there is a strong concordance between both methods.

## 2. Materials and Methods

### 2.1. Study Participants

This validation study was conducted with 16 healthy adult volunteers (6 women and 10 men). Their average age (±standard deviation, SD) was 24 ± 6 years with an average height of 174 ± 6 cm and an average body weight of 70.5 ± 10.1 kg.

The study was conducted in accordance with the Declaration of Helsinki and approved by the review board of the Prevention Center, TUM (#A2024004). Informed consent was obtained from all subjects involved in the study.

### 2.2. Study Devices

We compared two gait analysis systems. First, the CE-marked medical device software Orthelligent^®^ VISION (OPED GmbH, Valley/Oberlaindern, Germany) is a markerless pose estimation system based on AI and a computer vision model (MediaPipe BlazePose) that runs on mobile devices, capturing gait data only with the integrated RGB camera. For pose estimation, the system uses CNN to identify landmarks such as hips, knees, and ankles. The application automatically calculates relevant gait parameters for every step based on the skeletal tracking data and reports the parameters as means over all steps. This includes spatio-temporal parameters such as step length, walking speed, step frequency, cycle duration, and stance time. In addition, Orthelligent^®^ VISION analyzes symmetry parameters and gait phases. The results are provided after video capture and displayed both graphically and numerically, as well as by an automatically generated PDF report. Recorded data is processed and encrypted in the Microsoft Azure Cloud (Microsoft Corporation, WA, USA).

Second, the Vicon Motion Analysis System (Vicon Motion Systems Ltd., Oxford, UK) was used as the gold standard measurement system for motion analysis. The marker-based 3D motion capture system consisted of eight infrared cameras (T10, Vicon Motion Systems Ltd., Oxford, UK) and the Vicon Nexus software in combination with reflective markers (12 mm diameter, Prophysics AG, Kloten, Switzerland). These markers were placed bilaterally on anatomical landmarks in accordance with the conventional gait model (CGM 2.5 [16]).

### 2.3. Study Setup

**Figure 1:**
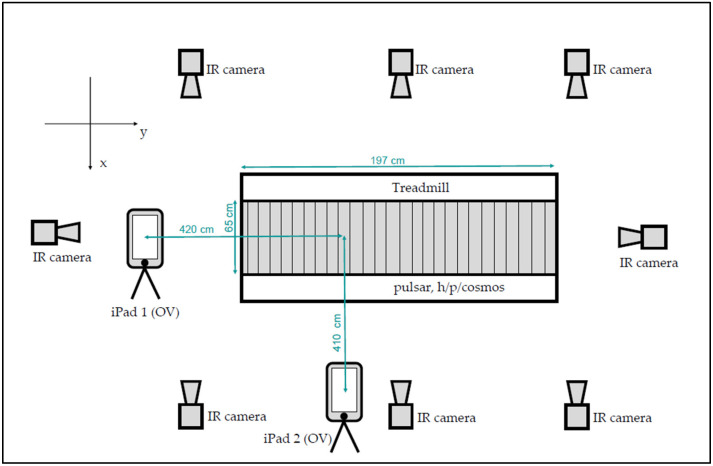
Setup for the validation study using a treadmill.

For the gait analyses, the volunteers were asked to walk on a treadmill (pulsar, h/p/cosmos sports and medical gmbh, Nussdorf-Traunstein, Germany). With both the markerless and the marker-based motion capturing systems, the participants’ motions were captured while walking at 4 km/h for one minute (Figure 1). A total of 52 consecutive steps (26 left, 26 right) were used for further analysis.

### 2.4. Markerless Motion Capturing

**Figure 2:**
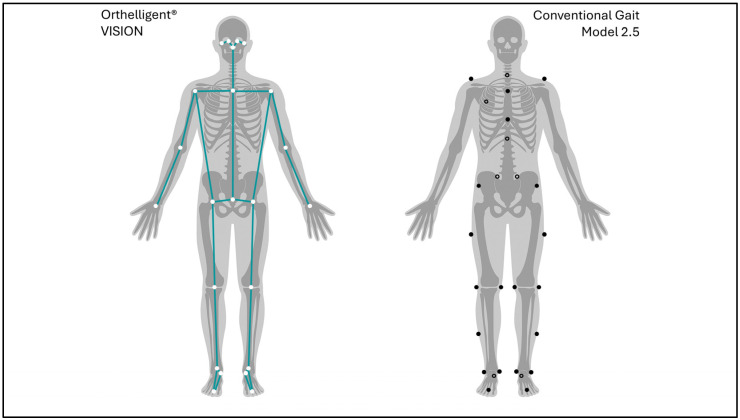
Left-hand side: Landmark positions for markerless motion capturing; and right-hand side: marker positions for 3D motion capture according to CGM 2.5. Filled black dots represent markers placed on the front side of the body, while black circles represent markers on the back side.

Two iPads (Apple Inc., Cupertino, CA, USA) with the installed Orthelligent^®^ VISION (OV) app were used to capture 2D videos at a sampling frequency of 30 fps. One was positioned 4.20 m in front of the treadmill and operated in upright mode to capture the subjects’ frontal plane. The second one was positioned 4.10 m on the left (in gait direction) of the treadmill and operated in horizontal mode to capture the subjects’ sagittal plane. The iPads’ cameras were positioned 1.30 m above ground level. Landmark positions include 33 human body key points (Figure 2). The model is based on 33 key points of the human body, while for kinematic calculations, the position of only 23 key points was used. The OV software does not require calibration.

### 2.5. Reference Standard: Marker-Based Motion Capturing

3D motion data were recorded using the marker-based Vicon Motion Analysis System (VIC; Vicon Motion Systems Ltd., Oxford, UK) at a sampling frequency of 100 fps. Reflective markers were placed bilaterally on anatomical landmarks in accordance with the CGM 2.5 [16]. The landmarks included the heel, toe, medial and lateral malleoli (ankle), shank, medial and lateral femoral condyles (knee), thigh, anterior superior iliac spine, and posterior superior iliac spine. Medial ankle and knee markers were used exclusively for static calibration (Figure 2). The capture volume was calibrated using a passive calibration wand (Vicon Motion Systems Ltd., Oxford, UK). For static subject calibration, participants stood upright on the treadmill performing a T-pose to ensure unobstructed visibility of all markers by the cameras.

### 2.6. Data Processing

3D kinematic data were captured and processed by using Vicon Nexus 2.15 (Vicon Motion Systems Ltd., Oxford, UK). After marker labeling and gap-filling, calculated marker positions were smoothed by a 15 Hz Woltring filter. Kinematic model output was derived from conventional gait model 2.5, including additional upper body markers on the thorax, clavicular, sternum, T2 vertebrae, T10 vertebrae, and right scapula [16,17].

With the markerless system, pose estimation landmarks from the frontal and lateral videos were processed by a custom smoothing Butterworth low-pass filter. Therefore, a second-order low-pass Butterworth filter was applied to OV data, using a cutoff frequency of 3 Hz and a sampling rate of 30 fps. This cut-off frequency was chosen as it is above the typical human gait frequency range (0.5–1.5 Hz). Thus, the motion patterns relevant for event detection are preserved while high-frequency noise is effectively attenuated. A similar approach was described for the automatic detection of gait events from kinematic data by O’Connor et al. [18].

For both systems, gait events were defined according to the approved literature and biomechanics-based methods [11]. The following were defined as gait events: the first contact of the foot with the ground as initial contact, and the moment when the toes leave the ground as toe-off.

As described before, the marker-based system recorded videos at 100 fps, while the markerless system recorded at 30 fps. The processed data sets were time-synchronized in accordance with gait events. For both systems, different coordinate systems were used. Tablets used for markerless gait analysis captured videos in full HD resolution (1920 × 1080 pixels). Body height in meters is used as a relative reference value to convert the pixel information. In contrast, the marker-based system provides coordinates in millimeters.

### 2.7. Analyzed Gait Parameters

The gait metrics described in Table 1 were calculated. These parameters capture essential aspects of motor control, balance, and interlimb coordination and are sensitive to both subtle and overt impairments of neuromuscular function. Their clinical relevance extends across multiple medical specialties, e.g., in neurology, for early detection and monitoring of diseases such as Parkinson’s disease, multiple sclerosis, and post-stroke hemiparesis; in orthopedics and rehabilitation medicine for functional information leading to treatment decisions following joint replacements or musculoskeletal injuries; or in geriatrics for the prediction of individual fall risk [15,19,20,21,22].

Gait symmetry was calculated using the following equation:

(1) $$Gait\;symmetry=\left(1-\left|\frac{2*\left(Xr-Xl\right)}{Xr+Xl}\right|\right)*100$$

For the lateral analysis, *Xr* and *Xl* represent the respective step lengths on the right-hand and left-hand sides, while for the frontal analysis, *Xr* and *Xl* are the variables for the respective step time on the right-hand and left-hand sides.

### 2.8. Statistical and Graphical Analysis

The processed data were further statistically analyzed with PYTHON using custom-written codes. We tested the continuous data for normal distribution by using the Shapiro–Wilk test, while the Breusch–Pagan test was used to test homoscedasticity as a requirement for further regression analysis. We evaluated the agreement between both measurements—marker-based 3D gait analysis (VIC, gold standard) and 2D markerless gait analysis (OV)—graphically and numerically as suggested by Koch & Spörl [23] and Grouven et al. [24]. For graphical analysis, scatter plots and Bland–Altman plots were created for each parameter [25,26]. The numerical analysis of agreement included calculation of the concordance correlation coefficient (CCC) according to Lin [27,28] for the agreement between the two measurement methods (see Table 2 for CCC’s heuristic meaning).

If variances are very low, the CCC is unlikely to reach a high value even if agreement within measurement systems is reasonable [29]. Above that, the coefficient of variation (CV) in percent is calculated for all metrics showing the level of spread of all parameters. Due to possible shifts in localization, scale, or both, we avoided the use of the Pearson correlation coefficient. All parameters are presented as mean ± SD unless otherwise stated.

## 3. Results

### 3.1. Gait Event Detection—Initial Contact and Toe-Off

As shown in Table 3, the number of gait events detected by the marker-based system is taken as the baseline for comparison. In the validation study, each subject took 52 steps, with 26 initial contacts for each of the left and the right leg. The markerless system was able to identify 100% of the initial contact events: 416 out of 416 initial contact events for the left leg and for the right legs, respectively. The markerless system also detected 98.7% of the toe-off events: 411 out of 416 toe-off events for each of the left and the right legs. In summary, with regard to initial contact and toe-off events, we found good overlaps for both systems.

Comparing the marker-based and the markerless system, the mean absolute error in identifying initial contact and toe-off events ranged from 3 ms to 15 ms, with 8 ms ± 11.49 ms and 5.21 ms ± 14.81 ms for the mean error ± SD for the left-side and the right-side initial contact events, respectively. The corresponding mean errors in identifying left-side and right-side toe-off events were 3.19 ms ± 14.01 ms and 6.81 ms ± 14.53 ms, respectively (Table 3).

### 3.2. Sagittal Analysis

The following seven gait parameters were important for the analysis from the lateral view: cadence, double support (mean of both sides), gait symmetry, gait variability (mean of both sides), stance time (mean of both sides), step length (mean of both sides), and step time (mean of both sides).

#### 3.2.1. Statistical Analysis of Sagittal Plane Gait Parameters

Table 4 summarizes the results of the statistical analysis that confirm a generally high level of agreement between the markerless and marker-based measurements. In particular, they showed an almost complete agreement for the gait parameters of cadence, stance time, step length, and step time with CCCs close to 1.0 and with low variances (CV). For double support and gait variability, the methods’ agreements were strong with CCCs of 0.734 and 0.624, while their agreement for gait symmetry was moderate. As mentioned before, very low variances, as with the parameter gait symmetry, resulted in a low CCC, although the agreement within the measurement systems was reasonable, which was supported by their similar means (97.0% and 96.0 SI).

#### 3.2.2. Graphical Analysis of Sagittal Plane Gait Parameters

**Figure 3:**
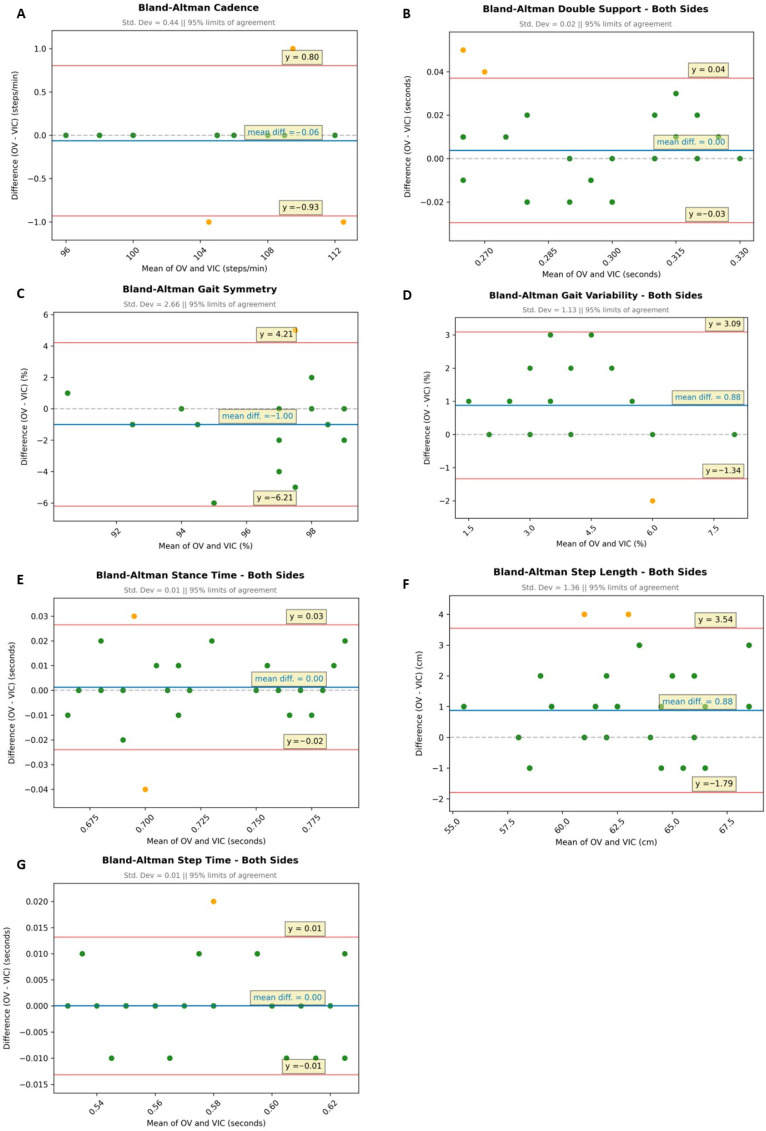
Comparison of markerless (OV) and marker-based (VIC) lateral gait analyses (N = 16) using Bland–Altman plots for the gait parameters: (**A**) cadence, (**B**) double support (mean of both sides), (**C**) gait symmetry, (**D**) gait variability (mean of both sides), (**E**) stance time (mean of both sides), (**F**) step length (mean of both sides), and (**G**) step time (mean of both sides); Bland–Altman plots: blue line: mean difference in a pair of variates; red lines: limits of agreement (LoA), ±1.96 × SD indicating the range in which 95% of the differences are to be expected; green dots: variates are within the LoA; yellow dots: variates are outside the LoA.

The Bland–Altman plots (Figure 3A–G) depict the difference between the two measurements (marker-based minus markerless) of the analyzed gait parameters (see Table 1) on the *y*-axis and their means on the *x*-axis, with each dot representing a pair of variates for one subject. Narrow limits of agreement (LoA, range for 95% of the differences) indicate great agreement.

In the lateral analysis, the two methods resulted in an excellent agreement with low scattering for the gait parameters of double support, stance time, and step time (Figure 3B,E,G), with a negligible mean difference of 0.00, a very small SD (0.01 and 0.02), as well as a narrow LoA (0.04 to −0.03). In addition, only one or two dots were outside the LoA, which might be due to measurement errors or single systematic offsets. In terms of cadence, the mean difference was only slightly higher (−0.06) with a larger but still low SD (0.44), wider LoA (0.80 to −0.93), and three dots outside the LoA (Figure 3A). The latter points to a good agreement of the measurements.

We found the highest mean differences (−1.0 to 0.88) for the metrics of gait symmetry, gait variability, and step length (Figure 3C,D,F). The respective SD ranged between 1.13 and 2.66 with a broader LoA (4.21 to −6.21). These findings suggest a slight systematic deviation. However, for each of the three parameters, only one dot was outside the 95% LoA, indicating a good agreement of the two measurements.

Of note, for some gait metrics, the Bland–Altman plots show fewer unique values (dots) than the total number of subjects. This means that several of the 16 subjects shared exactly the same data points.

**Figure 4:**
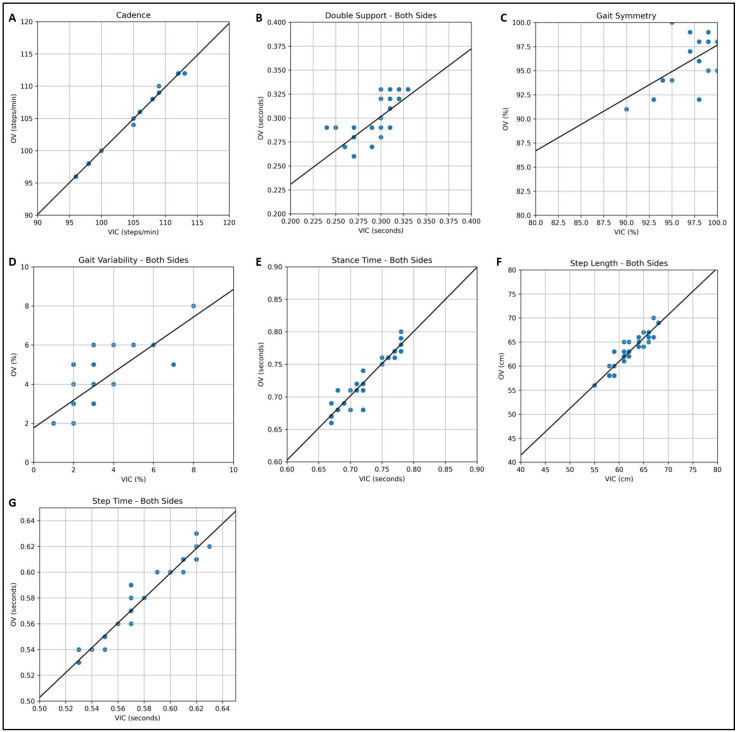
Comparison of markerless (OV) and marker-based (VIC) lateral gait analyses (N = 16) using scatter plots for the gait parameters: (**A**) cadence, (**B**) double support (mean of both sides), (**C**) gait symmetry, (**D**) gait variability (mean of both sides), (**E**) stance time (mean of both sides), (**F**) step length (mean of both sides), and (**G**) step time (mean of both sides).

The results of the two systems, VIC and OV, are also displayed in scatter plots (Figure 4A–G,) to confirm the correlations of the analyzed gait parameter. Similarly to the Bland–Altman plots, they show strong agreements for cadence (Figure 4A), stance time (Figure 4E), step length (Figure 4F), and step time (Figure 4G). A medium concordance was shown for the parameters of double support (Figure 4B) and gait variability (Figure 4D). However, the scatter plot indicates a low agreement for gait symmetry (Figure 4C).

### 3.3. Frontal Analysis

Deviating from the lateral analysis, in the frontal analysis a reduced set of five gait parameters were relevant: cadence, gait symmetry, stance time (mean of both sides), step width (mean of both sides), and step time (mean of both sides).

#### 3.3.1. Statistical Analysis of Frontal Plane Gait Parameters

As shown in Table 5, the two measurement methods resulted in almost complete agreement for nearly all gait parameters with CCCs between 0.805 and 0.993: cadence, stance time, step width, and step time. Their respective means were very similar. We only found a weak correlation for gait symmetry that might be due to the measurements’ low variances (CV_VIC_ = 2.870, CV_OV_ = 0.485) leading to a low CCC, as pointed out before. Their corresponding means differed slightly (Mean _VIC_ = 97.0% and Mean _OV_ 99.875%).

#### 3.3.2. Graphical Analysis of Frontal Plane Gait Parameters

**Figure 5:**
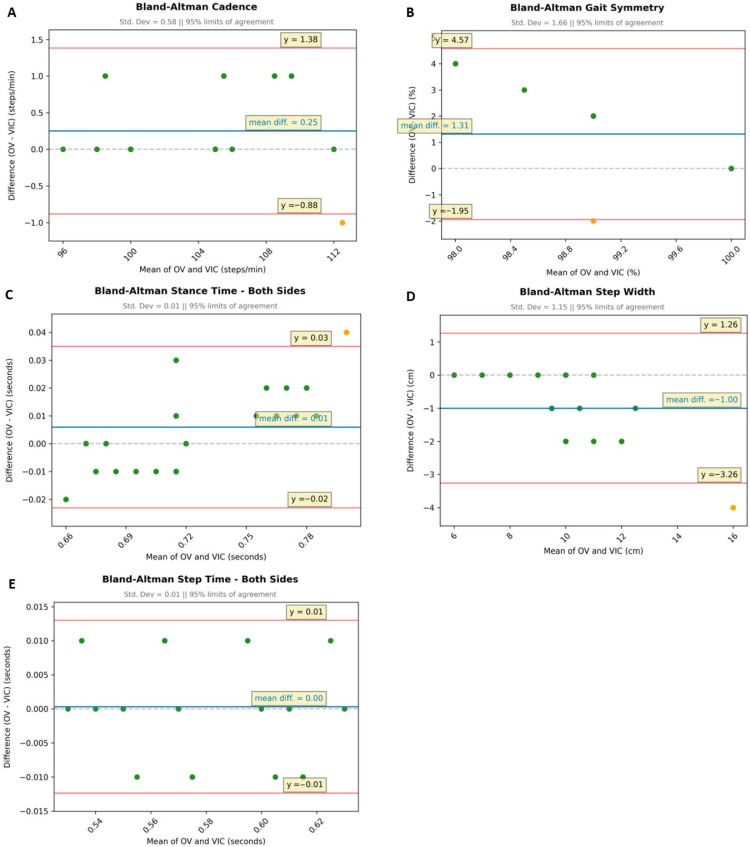
Comparison of markerless (OV) and marker-based (VIC) frontal gait analyses (N = 16) using Bland–Altman plots for the gait parameters: (**A**) cadence, (**B**) gait symmetry, (**C**) stance time (mean of both sides), (**D**) step width, and (**E**) step time (mean of both sides); Bland–Altman plots: blue line: mean difference in a pair of variates; red lines: limits of agreement (LoA), ±1.96 × SD indicating the range in which 95% of the differences are to be expected; green dots: variates are within the LoA; yellow dots: variates are outside the LoA.

In the frontal analysis, the Bland–Altman plots for the gait parameters of stance time and step time (Figure 5C,E) show a very strong agreement with mean differences of 0.01 or 0.00, respectively, as well as with low SD (both 0.01) and narrow LoA (0.03 to −0.02). The great agreement of the measurements was confirmed by no or only one dot outside the LoA.

For the gait parameter cadence (Figure 5A), we found a rather small mean difference (0.25) and a moderate SD (0.58), indicating a strong agreement of markerless and marker-based measurements. This was supported by a narrow LoA (1.38 to −0.88) and only one subject outside.

The mean differences for the parameters of gait symmetry and step with (Figure 5B,D) were slightly higher (1.31 and −1.00) with larger SDs (1.66 and 1.15) and LoA (4.57 to −3.26) than for the three other metrics. Nonetheless, each plot shows only one dot outside the respective LoA, which suggests a high agreement of the two methods.

**Figure 6:**
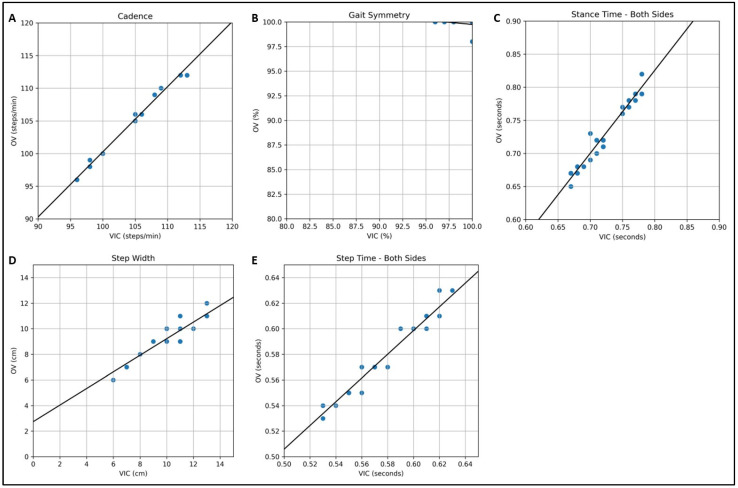
Comparison of markerless (OV) and marker-based (VIC) frontal gait analyses (N = 16) using scatter plots for the gait parameters: (**A**) cadence, (**B**) gait symmetry, (**C**) stance time (mean of both sides), (**D**) step width, and (**E**) step time (mean of both sides).

Similarly to the lateral analysis (Figure 3), the Bland–Altman plots for the frontal analysis (Figure 6, left-hand side) show that some subjects had the same data points.

In the frontal analysis, the scatter plots (Figure 6) show a strong agreement for the following four analyzed gait parameters: cadence, stance time (mean of both sides), step width, and step time (mean of both sides). In contrast, the scatter plot for gait symmetry confirms the results of the statistical analysis, indicating a weak agreement between the two methods.

## 4. Discussion

Markerless pose estimation offers new possibilities to extend the use of motion analysis. However, software-based medical devices for motion analysis must comply with high requirements on validity and reliability to serve as a basis for decision-making in clinical use. For this reason, the aim of the present pilot study was to evaluate the validity of OV for the assessment of spatio-temporal parameters in the sagittal and frontal planes during gait. Compared to the gold standard method—marker-based 3D motion analysis—OV achieved strong to almost complete concordance in the sagittal measurement of cadence, gait variability, step time, stance time, step length, and double support (CCC ≥ 0.624). OV also achieved a strong or almost complete concordance for the frontal plane parameters of cadence, step time, stance time, and step width (CCC ≥ 0.805).

While most of sagittal and frontal parameters have strong or almost complete concordance, the results of gait symmetry were conspicuous. Although, the mean differences between both systems are small (sagittal plane: mean difference _OV-VIC_ = 1%, frontal plane: mean difference _OV-VIC_ = 2.875%) there is only moderate concordance in the sagittal plane (CCC = 0.527) and weak concordance in the frontal plane (CCC = 0.108). The weak concordance might therefore result from the highly standardized setting with healthy participants. The small variance can be seen in Figure 6B where the 16 participants are represented by five dots in the top right corner (equal to 100% symmetry). This methodological artifact is also represented by low coefficients of variation in both planes [29]. Hence, variable walking velocities and overground walking, as well as healthy participants and patients with musculoskeletal or neurodegenerative disorders are needed to increase inter-individual variation in gait symmetry, which is needed for validity testing. Because of almost complete concordance relations in the underlying data—the step length in sagittal plane gait symmetry (CCC = 0.892) and step time in frontal plane gait symmetry (CCC = 0.975)—inherent limitations of pose estimation models are unlikely to be the reason for the weak concordance in gait symmetry.

The increasing number of pose estimation models and their further development also lead to raising evidence for the validity of these models [13,30,31,32,33,34]. The measurement of spatio-temporal parameters by pose estimation models typically demonstrates high validity [13,31,33,34]. For example, time parameters (stride/step/stance time) measured by marker-based motion capture and the markerless OpenPose algorithm achieved interclass-correlation-coefficients (ICCs) close to 1.0 in the studies by Yamamoto et al. (stride time ICC = 0.98 (95%CI [0.93, 0.99]; [33]), Matsuda et al. (stride time ICC = 0.932 (95%CI [0.832, 0.973]; [31]), and Stenum et al. (step time ICC = 0.955; [34]). For clinical relevance, it has to be considered that the absolute deviation in time parameters is dependent on the frame rates used. Capturing with 100 fps, these studies reported absolute time differences up to 10 ms. This is equivalent to one captured frame [31,33,34]. Using the video camera of common tablets, the markerless OV captures the gait with 30 fps, which is still suitable for walking analysis [35]. However, it was shown that for the mean absolute error in event detection (Table 3), as well as mean differences between both systems in time-based metrics (stance time, step time, double support time), their limits of agreement are below 40 ms or the one frame time window. A similar error range for event detection was also reported by Zeni et al., comparing 3D motion capture (60 fps) with synchronized force plate data (600 Hz) [36]. The limited frame rate affects the interpretation of the temporal parameters, due to their different time durations. The shorter the temporal parameter (e.g., double support compared to step time), the higher the relative system error, and vice versa. Consequently, walking speed also affects the relative system error in temporal parameters. Hence, the used version of OV is limited to gait analysis, since the relative error in event detection might be unacceptable in faster movements like running/jumping.

The high validity of the distance-based parameters step length (CCC = 0.982) and step width (CCC = 0.805) is also in line with recent studies [31,33,34]. Despite the high concordance, OV overestimated the step length by 1 cm compared to marker-based motion capture, while step width was underestimated by 1 cm. The absolute difference of 1 cm might not affect the clinical significance of the step length measured by OV. Conversely, due to its relative difference of almost 10%, the interpretation of step width must consider the individual case and purpose of the conducted gait analysis. Similarly to the temporal parameters, the spatial parameters are also limited by the hardware used. Recent camera sensors of modern tablet devices typically record in full-HD (1920 × 1080 pixel) resolution. Depending on the distance between camera and focused plain, one sensor pixel represents different distances, and thus calibration is necessary. Hereby, certain points have to be mentioned to ensure high quality video data, like (1) optimal camera–subject distance and image size depending on focal plane, (2) camera focus, (3) alignment of camera axis perpendicular to movement plane, and (4) proper lightning [35].

Knowing the strengths and weaknesses of pose estimation models in their application [14], gait and motion analysis have potential for a wide field of application in clinical use. For example, markerless motion analysis can be used for the documentation of the therapy outcome and rehabilitation progress after musculoskeletal injuries or orthopedic surgeries like joint replacements [14,37], for the clinical assessment of motor function in patients with neurodegenerative diseases [14,15], or for safety measures such as fall prevention [14,38]. These application fields can be addressed by the markerless quantification of single joint movements and complex whole-body movements like the stand up and go test [14]. Nevertheless, to implement markerless movement analysis in clinical practice, the software as a medical device has to overcome the following barriers: usability, presentation of relevant and clear results, the needed hardware and software, and present validation [14]. As a tablet-based medical device software, the strengths of the certified markerless gait analysis software OV are particularly evident in overcoming these barriers due to ease of use, reduced resource requirements, and immediate availability of results [8,12,15]. With ongoing development, computer vision machine learning approaches might be able not only to detect movement patterns but also classify them to certain pathological conditions. For this, Hassine et al. [39] trained machine learning algorithms for the classification of knee osteoarthritis in different severity stages based on kinematic data evaluated by Mediapipe pose estimation model. Their approach was effective in the classification of knee osteoarthritis even though a greater data set is needed to improve classification and generate individual treatment plans [39].

The limitations of the pilot validation study should be acknowledged. This study was designed as pilot validation of spatio-temporal gait analysis in a highly standardized and controlled setting including 16 healthy participants walking on a treadmill with a fixed speed. This design led to small absolute differences between VIC and OV with strong or almost complete concordance. Regarding gait symmetry, the setting could limit the inter-individual variation, resulting in hardy interpretable CCC in both planes. Thus, the results of this pilot study are limited in generalizability but may serve as scientific basis for further investigations on validation in patients with musculoskeletal injuries and diseases, as well as neurodegenerative disorders, during overground walking. Further studies should also test the reliability of the system, addressing inter-rater and intra-rater reliability, as well as performance under diverse environmental conditions (e.g., varying illumination, different backgrounds). This information is needed to better assess the system’s applicability in routine clinical settings.

## 5. Conclusions

This pilot validation study provides evidence for the clinical applicability of the CNN-based, markerless gait analysis system OV as an alternative to conventional, marker-based 3D motion capture systems in clinical routine.

Based on the data presented, OV can be used as a valid measurement system for clinical gait analysis. Its ease of use, cost efficiency, and robust performance are beneficial for the digital transformation of musculoskeletal and neurological care. Thus, OV has the potential to implement gait analysis in clinical decision-making, injury prevention, and rehabilitation.

Especially in an increasingly digitalized and resource-limited healthcare system, OV offers a forward-looking solution for objective, efficient, and user-friendly motion analysis. In the long run, this enables possibilities for patient-centered care and research in real-world settings, as the possibility of performing gait analyses outside of specialized laboratories considerably expands the range of applications for markerless systems.

## Figures and Tables

**Table 1:** Analyzed gait parameters, the general calculation methods for both systems, and their clinical relevance according to evidence [19,20,21,22].

Parameter [Unit]	Description	Calculation	Clinical Relevance
Gait symmetry [%]	The gait symmetry indicates how symmetrical the left and right step length is. The higher the value, the better.	Calculated using step length differences between sides (lateral).	Early marker for motor dysfunctions or insidious neurological diseases.
Cadence [steps/min]	The gait cadence indicates the total number of steps taken within a minute during the analysis.		Early biomarker for neurodegenerative diseases and predictor for fall risk, especially in geriatric populations.
Double support left/right [%]	The gait double support indicates proportion of time that both feet of the patient are on the ground during the analysis.	Calculated in percentage and shows the part where both feet are on the ground.	Highly sensitive gait parameter for the detection of asymmetric gait patterns, as they occur in many neurological and orthopedic diseases.
Gait variability left/right [%]	The gait variability indicates step-to-step length fluctuation of left/right leg during the analysis.	Derived from the standard deviation and mean of the step length.	Early marker for neurodegenerative diseases (Parkinson’s, dementia) and fall risk indicator.
Step length Left/Right [cm]	The gait step length indicates the average distance between the point of initial contact of the left/right foot and the point of initial contact of the other foot during the analysis.	Distance between the toes of the left and right leg during the initial contact.	Central gait parameter that is used both in diagnostics and for progress assessments.
Step time left/right [s]	The gait step time indicates the average time elapsed from initial contact of the left/right foot to initial contact of the other foot during the analysis.	Difference between the gait events terminal swing and terminal stance.	Central gait parameter that is used both in diagnostics and for progress assessments.
Stance time left/right [%]	The gait stance time indicates the average percentage of time during which the left/right foot is in contact with the ground during the analysis.	Calculated in percentage of a gait cycle using the pre-swing and the initial contact.	Another central gait parameter used in diagnostics, progression assessment, fall risk assessment, and therapy management.
Step width [cm]	The step width indicates the average frontal plane distance between the point of initial contact of the left/right foot and the point of initial contact of the other foot during the analysis.	The distance between left and right heel in frontal plane.	Assessment of stability and balance control.

**Table 2:** Concordance correlation coefficients (CCCs) and their heuristic meaning.

Concordance Correlation Coefficient	Heuristic Meaning
<0.10	none
0.10–0.40	weak
0.41–0.60	moderate
0.61–0.80	strong
0.81–1.00	almost complete

**Table 3:** Identification of key gait events (initial contact and toe-off) with a marker-based motion capture system (VIC) versus markerless pose estimation analysis (OV).

Gait Event	Leg	Mean Absolute Error ± SD (VIC—OV)
Initial contact	Left	8 ms ± 11.49 ms
	Right	5.21 ms ± 14.81 ms
Toe-off	Left	3.19 ms ± 14.01 ms
	Right	6.81 ms ± 14.53 ms

**Table 4:** Mean, standard deviation (SD), concordance correlation coefficients (CCCs) including their respective heuristic meaning, and coefficient of variation (CV) with a marker-based motion capture system (VIC) versus markerless pose estimation analysis (OV), lateral analysis (N = 16).

Metric	Device	Mean	SD	CCC [Heuristic Meaning]	CV [%]	Limits of Agreement
Cadence [steps/min]	VIC	104.312	±5.009	0.996 [almost complete]	4.802	0.08/−0.93
	OV	104.250	±4.969		4.766	
Gait symmetry [%]	VIC	97.000	±2.784	0.527 [moderate]	2.870	4.21/−6.21
	OV	96.000	±2.716		2.829	
Gait variability [%]	VIC	3.031	±1.531	0.624 [strong]	50.494	3.09/−1.34
	OV	3.906	±1.487		38.057	
Step time [s]	VIC	0.576	±0.029	0.974 [almost complete]	5.036	0.01/−0.01
	OV	0.576	±0.029		4.980	
Stance time [s]	VIC	0.723	±0.038	0.947 [almost complete]	5.293	0.03/−0.02
	OV	0.724	±0.040		5.512	
Step length [cm]	VIC	62.188	±3.292	0.892 [almost complete]	5.294	3.54/−1.79
	OV	63.062	±3.473		5.507	
Double support [s]	VIC	0.293	±0.024	0.734 [strong]	8.198	0.04/−0.03
	OV	0.296	±0.023		7.677	

**Table 5:** Mean, standard deviation (SD), concordance correlation coefficients (CCCs) including their respective heuristic meaning, and coefficients of variation (CV) with a marker-based motion capture system (VIC) versus markerless pose estimation analysis (OV), frontal analysis (N = 16).

Metric	Device	Mean	SD	CCC [Heuristic Meaning]	CV [%]	Limits of Agreement
Step width [cm]	VIC	10.625	±2.666	0.805 [strong/almost complete]	25.095	1.26/−3.26
	OV	9.625	±1.833		19.043	
Step time [s]	VIC	0.576	±0.029	0.975 [almost complete]	4.951	0.01/−0.01
	OV	0.577	±0.028		4.777	
Stance time [s]	VIC	0.723	±0.038	0.936 [almost complete]	5.274	0.03/−0.02
	OV	0.728	±0.050		6.850	
Cadence [steps/min]	VIC	104.312	±5.009	0.993 [almost complete]	4.802	1.38/−0.88
	OV	104.562	±5.025		4.805	
Gait symmetry [%]	VIC	97.000	±2.784	0.108 [weak]	2.870	4.57/−1.95
	OV	99.875	±0.484		0.485	

## Data Availability

All data, tables, and figures presented in this manuscript are original. Further inquiries can be directed to the corresponding author.

## Abbreviations

The following abbreviations are used in this manuscript:

AI	Artificial intelligence
CCC	Concordance Correlation Coefficient
CNN	Convolutional Neural Network
CV	Coefficient of Variation
ICC	Interclass-Correlation-Coefficient
LoA	Limit of agreement
ML	Machine learning
OV	Orthelligent^®^ Vision (markerless system)
SD	Standard deviation
VIC	Vicon (marker-based system)
